# A Rare Case of Coronary Stent Thrombosis in the Modern Era

**DOI:** 10.7759/cureus.25207

**Published:** 2022-05-22

**Authors:** Khandakar M Hussain, Ashish Jain, Rahul Prakash Rane, Yazeed G Sweedan, Asna Shahab, Md Didar Ul Alam, K. M. Anwar Hussain

**Affiliations:** 1 Internal Medicine, Conemaugh Memorial Medical Center, Johnstown, USA; 2 Cardiology, Conemaugh Memorial Medical Center, Johnstown, USA

**Keywords:** primary percutaneous coronary intervention, stemi, percutaneous coronary intervention, drug-eluting stents, coronary stent thrombosis

## Abstract

In patients with acute coronary syndrome or obstructive coronary artery disease, stents, especially drug-eluting stents (DESs), are used for percutaneous coronary interventions (PCI). DES prevents abrupt closure of the stented artery. Stent thrombosis is an uncommon but serious complication of PCI, especially with the recent advancement of stent technology. We present a case of a 75-year-old male who initially suffered a non-ST segment elevation myocardial infarction (NSTEMI) treated appropriately with PCI and subsequently developed stent thrombosis after 10 days of initial stent placement. He then underwent emergent repeat PCI with successful replacement of stents overlapping previous stents. The patient did well following the procedure. His clopidogrel was changed to a more potent antiplatelet ticagrelor. He remained stable throughout the hospital stay and was discharged home without any further complications following the next 90 days.

## Introduction

Stent thrombosis is the most feared complication of coronary stent placement; however, it is known but rare during the modern era of drug-eluting stents (DESs) [1]. Most commonly it presents as acute myocardial infarction (MI). Treatment for stent thrombosis requires emergent repeat percutaneous coronary intervention (PCI) [2]. Despite compliance with dual antiplatelet therapy, our patient suffered stent thrombosis requiring emergent PCI, which potentially saved his life.

## Case presentation

**Figure 1:**
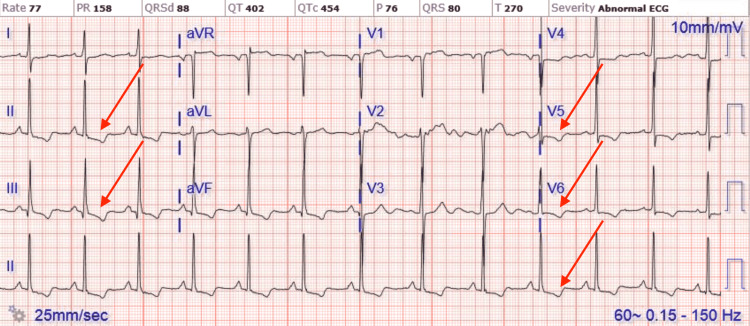
ECG of initial NSTEMI showing T wave inversion in leads II, III, aVF, and V4-6. ECG, electrocardiogram; NSTEMI, non-ST segment elevation myocardial infarction

**Figure 2:**
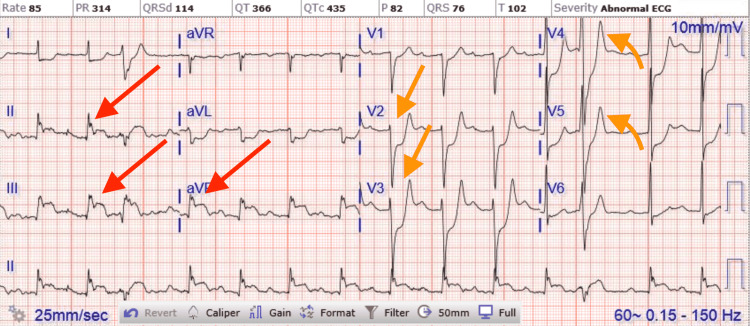
ECG on second admission showing acute ST segment elevation in inferior leads II, III, and aVF, with ST depression and peaked T wave on anterior leads causing inferior posterior myocardial infarction. ECG, electrocardiogram

**Figure 3:**
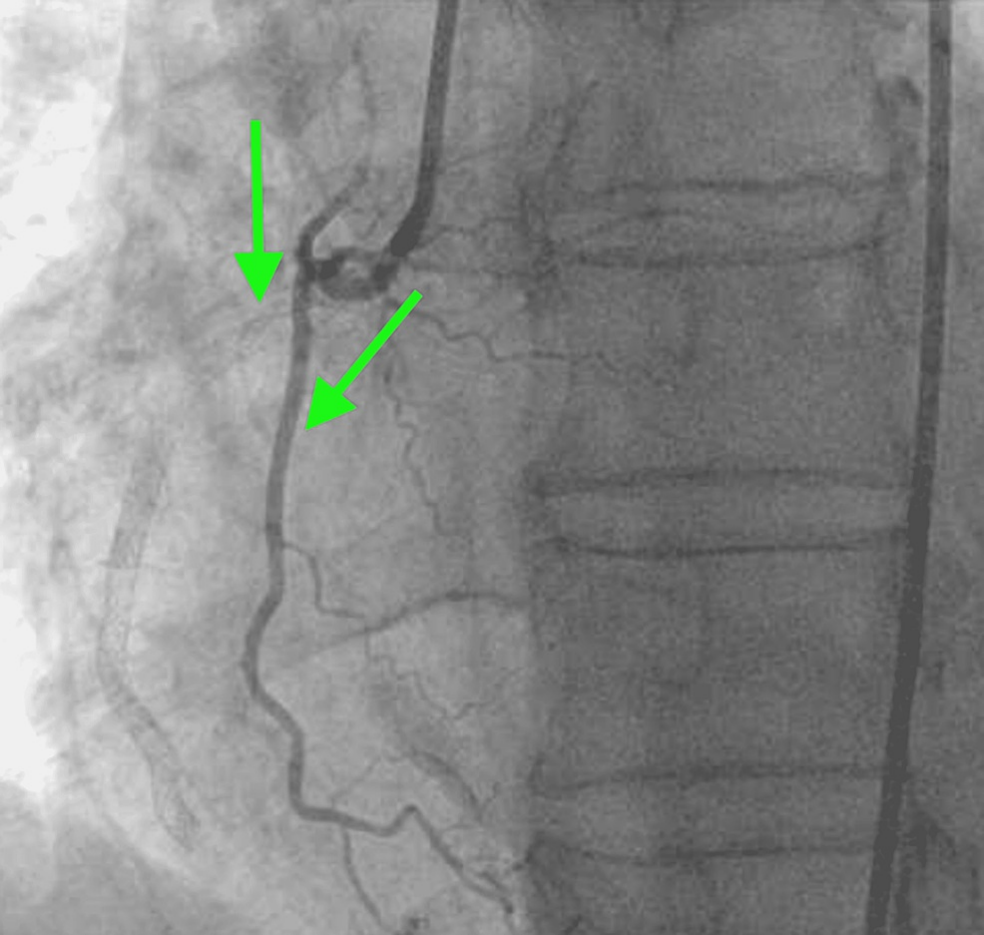
Proximal RCA stent thrombosis with very proximal RCA branch giving retrograde blood flow to distal, mid, and proximal RCAs with fade contrast filling. RCA, right coronary artery

**Figure 4:**
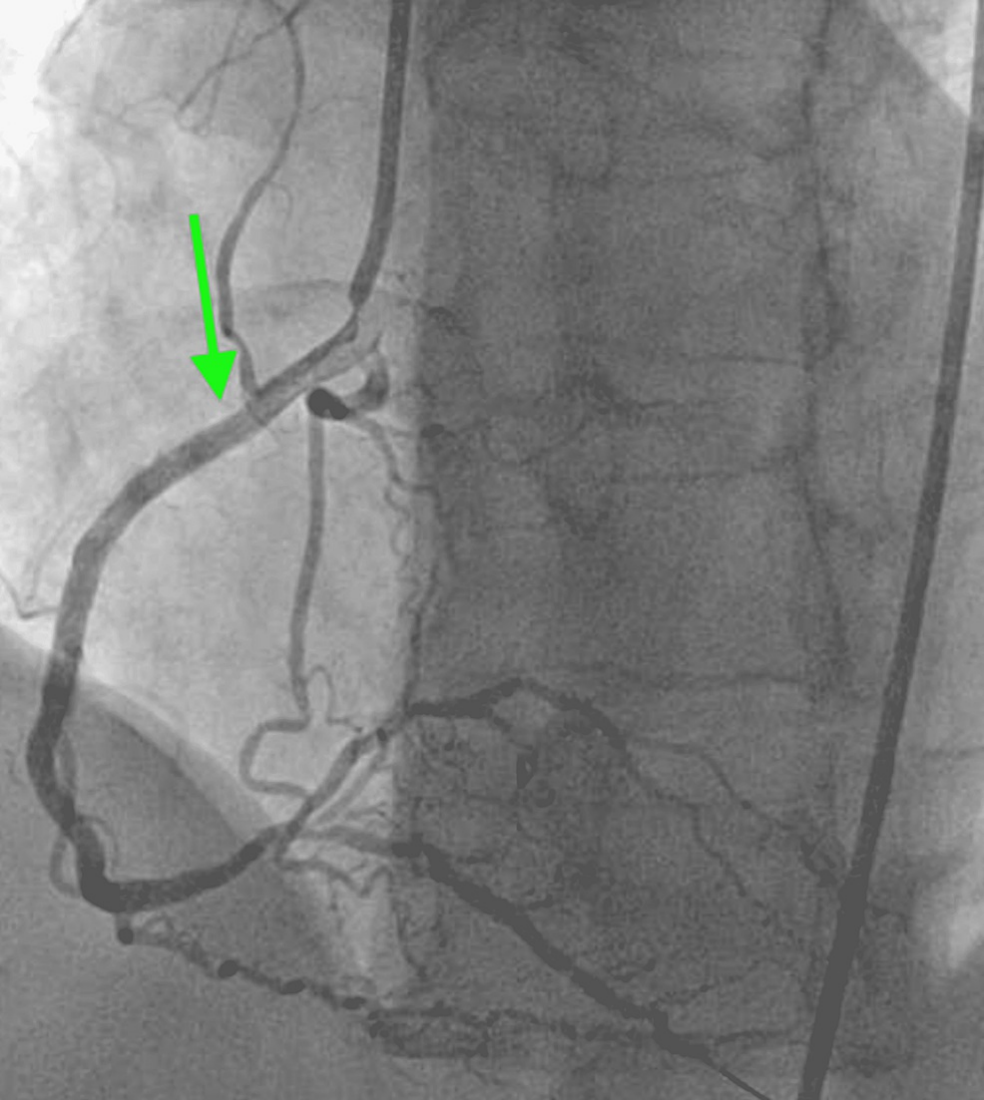
Successful restenting of the occluded proximal RCA with TIMI grade III flow. RCA, right coronary artery; TIMI, thrombolysis in myocardial infarction

**Figure 5:**
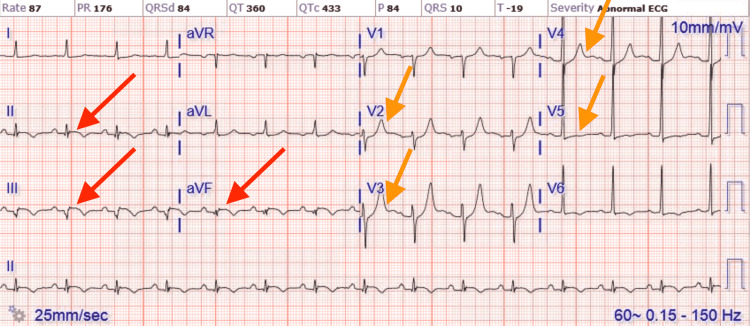
ECG after 12 hours of new stents placement in RCA and mLCX showed an evolutionary pattern of acute inferior posterior wall myocardial infarction with normalization of anterior wall changes. ECG, electrocardiogram; RCA, right coronary artery; mLCX, mid left circumflex artery

A 75-year-old male with a past medical history significant for uncontrolled hypertension, cigarette smoking of one packet daily, chronic obstructive pulmonary disease (COPD), and aspirin allergy presented to the emergency department with substernal chest pain and shortness of breath. An electrocardiogram (ECG) showed T wave inversion in leads II, III, aVF, and V4-6 (Figure 1). The chest X-ray was unremarkable. Initial high-sensitivity troponin (HST) was 400 ng/L (institutional normal HST range: 0.00-53.00 ng/L). He was not given aspirin on admission due to fear of severe anaphylaxis and was started on an unfractionated heparin drip. The patient received an intravenous heparin bolus of 60 units/kg followed by 12 units/kg/h continuous heparin infusion. His initial activated partial thromboplastin (aPTT) time was 44 seconds after the bolus followed by 55 seconds during one hour of continuous infusion (institutional normal aPTT is 23-35 seconds). Heparin infusion was continued till the PCI with aPTT was maintained for around 60 seconds. He then underwent PCI, which showed 90% stenosis of the mid-left circumflex artery (mLCX) and 90% stenosis of the proximal right coronary artery (RCA). Percutaneous transluminal coronary angioplasty (PTCA) and PCI of mLCX and RCA were performed. Intravascular ultrasound (IVUS) demonstrated no stent under expansion or distal edge dissection, and plaque burden was covered. Due to clot burden, the RCA lesion was predilated using a 2.5 x 20 mm compliant balloon and then stented with a 2.75 x 38 mm stent followed by a 3.0 x 38 mm stent just proximal and overlapping with the first stent. Latest generation resolute onyx DES was used. Thrombolysis in myocardial infarction (TIMI) III flow was achieved distally. The patient was loaded with clopidogrel 600 mg in the Cath Lab and carefully started on aspirin the day after the procedure due to fear of allergic reaction. However, the patient tolerated both aspirin and clopidogrel well. He was also given metoprolol tartrate and lisinopril. He did not receive ticagrelor due to active wheezing and shortness of breath present on admission due to COPD. Prasugrel was also not an option due to the age of 75 with a high risk of intracranial bleeding. His COPD was treated with a bronchodilator and steroid, with complete resolution of his symptoms. An echocardiogram of the heart showed an ejection fraction of 55% to 60%. The patient was subsequently discharged home in a stable condition on a dual antiplatelet therapy (DAPT). He came back to the hospital on day 10th after initial admission with acute substernal chest pain. Chest pain started around 10 am in the morning when he called emergency medical services (EMS). EMS performed an ECG around 10:20 am prehospital, which showed acute ST-segment elevation in inferior leads II, III, and aVF, with ST depression and peaked T wave on anterior leads causing inferior posterior MI (Figure 2). More prominent ST elevation on lead III suggests proximal RCA stenosis. Cardiac catheterization lab was activated, and the patient underwent emergent PCI and showed a completely occluded mLCx artery at the site of the stent with in-stent thrombosis, also completely occluded, of proximal RCA (Figure 3). A resolute onyx 2.75/22 mm stent was placed in the proximal RCA overlapping the prior stent and extending into the proximal part of the proximal RCA (Figure 4). Also, mLCx was successfully treated with a DES. TIMI grade III flow was achieved without any complications. His proximal left anterior descending artery was 50% stenosed and his posterior descending artery was 20% stenosed. IVUS was performed, which failed to show any under expansion of the stent or distal edge dissection. A loading dose of ticagrelor 180 mg was given, clopidogrel was discontinued, and aspirin was continued. Post-procedure ECG after 12 hours showed an evolutionary pattern of acute inferior wall MI after the placement of new stents in RCA and mLCX with normalization of anterior wall changes (Figure 5). During the initial echocardiogram, no regional wall motion abnormality was seen. During the second admission, an echocardiogram was not performed; however, left ventriculography during PCI was performed, which showed an ejection fraction of 50% and mildly hypokinetic basal inferior segment of the left ventricle. He was discharged home after 48 hours in a stable condition. The patient was closely followed up in the outpatient clinic for the following 90 days. He stopped smoking afterward and was compliant with medications. The patient was counseled about a possible need for coronary artery bypass graft (CABG) with open heart surgery if he develops recurrent stent thrombosis. He was agreeable for CABG if needed.

## Discussion

Stent thrombosis is defined by Academic Research Consortium (ARC) as angiographically confirmation of a thrombus that originates in the stent or in the segment 5 mm proximal or distal to the stent with or without vessel occlusion, which is associated with acute onset of ischemic symptoms at rest or ECG sign of acute ischemia [3]. The most common presentation of stent thrombosis includes ST-elevation myocardial infarction (STEMI) 60%, 23% non-STEMI (NSTEMI), and 17% unstable angina [4]. Risk factors for stent thrombosis are discussed in Table 1 [5-9].

**Table 1:** Risk factors for stent thrombosis ACS, acute coronary syndrome; CAD, coronary artery disease

Risk factors for stent thrombosis
ACS and proximal left anterior descending coronary artery lesion (for early stent thrombosis)
side-branch stenting, diabetes mellitus, and end-stage kidney disease
Incomplete stent expansion
Greater stent length
Residual plaque burden and small stent area on intracoronary ultrasound
Small vessel caliber
Residual thrombus or persistent dissection after stent placement.
Inflow or outflow obstruction
Subtherapeutic periprocedural anticoagulation
Emergent stent placement
Post-procedure TIMI flow grade < 3
CAD ≥ 50% proximal of culprit lesion
Treatment of bifurcation lesions
Multivessel disease, as shown in the SYNTAX trial
High treatment (oral antiplatelet therapy) platelet reactivity, including polymorphisms in the genes controlling hepatic enzymes involved in the metabolism of clopidogrel
The use of clopidogrel rather than prasugrel or ticagrelor in patients with ACS
Current smoking
No aspirin at the time of the procedure
Cocaine use
Left ventricular dysfunction

Stent thrombosis should be considered in any patient who presents with acute coronary syndrome (ACS) after stent placement, and there is no information from the history, physical examination, or ECG that can be used to clearly discriminate between stent thrombosis and ACS due to ischemia from a lesion(s) not related to prior stent placement. In the presence of ST-elevation or other changes suggestive of target (stented) vessel ischemia, the suspicion of stent thrombosis should be higher. These events should be classified as probable stent thrombosis by the ARC criteria unless angiography confirms another culprit. Patients with STEMI due to stent thrombosis should be managed with primary PCI.

Poor compliance with the DAPT may be the primary cause of the development of stent thrombosis. Nonresponsiveness to clopidogrel may be an important contributing factor in many cases and should be considered. Nonresponsiveness to clopidogrel is defined as inadequate antiplatelet response to clopidogrel. It can happen due to noncompliance with clopidogrel; however, our patient was very compliant with his medication after discharge. Other potential causes are variation in clopidogrel metabolism, interaction with other drugs such as proton pump inhibitors and calcium channel blockers, and genetic variation. A definitive diagnosis of clopidogrel treatment failure requires laboratory testing. P2Y12 assay can be used to assess the degree of platelet receptor inhibition. VerifyNow has been used in the main prospective clinical trials of personalized antiplatelet therapy [10]. With the VerifyNow P2Y12 assay, values > 208 PRU are required for the diagnosis. Our patient's P2Y12 assay was 215, which was performed during the second admission, which suggests clopidogrel nonresponsiveness.

The optimal approach to antiplatelet therapy after stent thrombosis in patients who demonstrate clopidogrel nonresponse is unknown. In patients who present with stent thrombosis while on clopidogrel, it is likely that the risk of recurrent stent thrombosis is increased. While no trials of alternate antiplatelet regimens, such as switching to prasugrel or ticagrelor have been performed in these patients, it is an option. The minimum duration of DAPT should be one year, with longer courses for patients who are tolerating such therapy. Among patients with an ACS and angiographically confirmed (definite) stent thrombosis in a pooled analysis, the 30-day rate of MI was 32%, with 30-day mortality rates after stent thrombosis of 7% for angiographically confirmed and 19% for clinically identified stent thrombosis for bare metal stents and 15% for DESs based on pooled clinical trial data [11,12].

Despite our strong recommendations, the patient continued to smoke one packet of cigarettes daily following the initial stent placement. Potential causes of our patient’s stent thrombosis include continued cigarette smoking, inability to use aspirin during or before the initial PCI, clopidogrel nonresponse, high clot burden, proximal lesion, and multivessel diseases.

## Conclusions

Tremendous advancement in coronary stent technology has been achieved in the past decade. The incidence of stent thrombosis has been very low in recent years. However, it still carries a significant impact on morbidity and mortality. Most patients present with ACS with ST-segment elevation on the ECG, which needs emergent PCI. In addition, these patients could be better served with higher potent antiplatelet therapy including prasugrel or ticagrelor. Clopidogrel nonresponsiveness is a serious issue in patients who have a contraindication for the use of prasugrel or ticagrelor. It is reasonable to test those patients with a P2Y12 assay. Also, strong counseling should be provided about smoking cessation and medication compliance. Early recognition and prompt treatment can prevent significant mortality and morbidity.
